# Learning to remain: Silence, suffering, and the hidden curriculum of care

**DOI:** 10.1017/S1478951526103071

**Published:** 2026-06-19

**Authors:** Renato Lorenzon Maisano

**Affiliations:** Ribeirão Preto Medical School, University of São Paulo, Ribeirão Preto, São Paulo, Brazil

No one had taught us what to do with silence.

In medical education, silence is often treated as absence: absence of breath sounds, absence of response, and absence of measurable improvement. We are trained to detect, classify, and interpret. A pulse can be counted; oxygen saturation can be monitored; renal failure can be staged; and pain can be scored. But the silence that gathers around a frightened patient, an exhausted relative, or a student suddenly aware of his own helplessness does not easily fit into a chart.

This is perhaps why it unsettles us.

I entered medicine carrying an assumption I had not yet examined: that clinical training would teach me not only how to act, but how to be present. What it taught me first, instead, was the particular psychological demand of becoming a physician – the expectation that one should be close enough to suffering to understand it, but distant enough to function inside it.

The contradiction is rarely stated. It is learned.

Medical students are formally taught empathy, communication, and patient-centered care. Informally, however, we also learn the grammar of emotional containment. We learn which questions can be asked in public and which must be carried privately. We learn to keep moving after encounters that should have stopped us. We learn, very early, that competence is often confused with composure.

This is the hidden curriculum of suffering. It does not appear as a lecture but as atmosphere. It is present when exhaustion is praised as dedication, when emotional reaction is treated as inexperience, and when asking for help feels like admitting that one is not suited for medicine. Hafferty and Franks described the hidden curriculum as a powerful force in the formation of physicians; in the emotional life of training, its lessons may be especially durable (Hafferty and Franks 1994).

I remember a room in which nothing urgent was happening. There was no dramatic emergency, no hurried intervention, and no monitor announcing a crisis. The patient had been made comfortable. A relative remained nearby, speaking softly, as if language itself had to be careful. What made the room difficult was not the absence of care but the recognition that care had changed direction. For a student accustomed to imagining medicine as action, the scene felt almost destabilizing.

Nothing needed to be conquered.

Something needed to be witnessed.

Palliative care understands this better than many areas of medicine because it does not hide from the limits of cure. It refuses the false conclusion that when disease can no longer be reversed, medicine has nothing left to offer. Instead, it shifts attention from disease alone to the person who continues to experience fear, pain, memory, dependency, love, and meaning.

This reorientation is ethically powerful, but psychologically demanding. For the clinician, and especially for the student, it requires learning that presence is not passivity. Listening is not inaction. Remaining beside a person without pretending to control what cannot be controlled is not failure. It may be one of the most mature forms of care.

Cassell argued that suffering concerns threats to the integrity of the person, not merely the progression of disease (Cassell 1982). This distinction matters because it exposes the insufficiency of a purely technical response to human distress. The body can be treated while the person remains unseen. Symptoms can be reduced while abandonment persists. A chart can be complete while the room remains empty in the ways that matter most.

This is where silence becomes clinical.

There are silences that indicate neglect, avoidance, or fear. There are also silences that protect dignity. The difference is not found in the silence itself, but in the quality of presence around it. A clinician who remains attentive, even without words, can communicate that the person before them has not been reduced to prognosis, bed number, or problem list.

For students, however, such presence is not automatic. It must be learned, and it must be protected.

The emotional costs of medical training are now widely recognized. Burnout, depersonalization, anxiety, and exhaustion are not marginal problems within the profession; they are structural warnings. West and colleagues have described physician burnout as a condition with consequences for clinicians, patients, and health systems (West et al. 2018). Yet the language of burnout can sometimes obscure the quieter process that precedes it: the gradual narrowing of one’s emotional life in order to survive constant exposure to vulnerability.

Many students do not collapse. They adapt. They become efficient, organized, and outwardly functional. They learn to speak fluently about suffering while becoming less able to feel what the word means. They become skilled at appearing fine.

This adaptation may be rewarded, but it is not neutral.

When emotional erasure is mistaken for professionalism, the clinician loses something essential. Patients do not need unbounded emotional exposure from those who care for them, but they do need the sense that they are being met by another human being. Professional boundaries should make care safer; they should not make care cold.

There is a difference between emotional regulation and emotional disappearance.

Narrative medicine offers one possible resistance to this disappearance. Charon described narrative competence as the capacity to recognize, absorb, interpret, and be moved by the stories of illness (Charon 2006). The phrase “be moved” is important. It suggests that being affected is not the enemy of clinical practice. The task is not to avoid being moved but to learn what to do with that movement: how to transform it into attention, humility, and ethical presence rather than avoidance or collapse.

This is not sentimentalism. It is clinical formation.

If medicine wants professionals capable of accompanying suffering without abandoning either the patient or themselves, reflective spaces cannot remain optional ornaments in the curriculum. Mentorship, narrative writing, supervised discussion of difficult encounters, and psychologically safe conversations about distress should be understood as part of patient care. A student who learns only to suppress vulnerability may become technically competent, but emotionally unavailable. A professional who cannot recognize their own suffering may struggle to perceive it fully in others.

The point is not to romanticize suffering. Suffering does not purify medicine. It can break people, distort institutions, and harden compassion into performance. The point is that the encounter with suffering is unavoidable, and silence around its impact on clinicians is itself a form of educational failure.

Perhaps the task is more modest and more difficult: to teach future physicians how to remain.

To remain without mistaking helplessness for uselessness.

To remain without turning away from the person when the disease can no longer be mastered.

To remain without allowing professional identity to depend on emotional numbness.

There will always be moments in medicine when knowledge reaches the edge of what it can repair. At that edge, the clinician’s presence is tested. Not because presence cures, but because abandonment wounds.

The patient may not remember every explanation. The family may not remember every result. But they may remember whether someone stayed long enough for their fear to be seen.

And the student, if properly guided, may learn that this too is medicine.
